# Temperature Influences the Interaction between SARS-CoV-2 Spike from Omicron Subvariants and Human ACE2

**DOI:** 10.3390/v14102178

**Published:** 2022-09-30

**Authors:** Shang Yu Gong, Shilei Ding, Mehdi Benlarbi, Yaozong Chen, Dani Vézina, Lorie Marchitto, Guillaume Beaudoin-Bussières, Guillaume Goyette, Catherine Bourassa, Yuxia Bo, Halima Medjahed, Inès Levade, Marzena Pazgier, Marceline Côté, Jonathan Richard, Jérémie Prévost, Andrés Finzi

**Affiliations:** 1 Centre de Recherche du CHUM, Montreal, QC H2X 0A9, Canada; 2 Department of Microbiology and Immunology, McGill University, Montreal, QC H3A 2B4, Canada; 3 Département de Microbiologie, Infectiologie et Immunologie, Université de Montréal, Montreal, QC H2X 0A9, Canada; 4 Infectious Disease Division, Department of Medicine of Uniformed Services, University of the Health Sciences, Bethesda, MD 20814-4712, USA; 5 Department of Biochemistry, Microbiology and Immunology, Centre for Infection, Immunity and Inflammation, University of Ottawa, Ottawa, ON K1H 8M5, Canada; 6 Laboratoire de Santé Publique du Québec, Institut National de Santé Publique du Québec, Sainte-Anne-de-Bellevue, QC H9X 3R5, Canada

**Keywords:** COVID-19, SARS-CoV-2, Spike glycoprotein, RBD, temperature, Omicron, BA.4, BA.5, ACE2 affinity, variant of concern

## Abstract

SARS-CoV-2 continues to infect millions of people worldwide. The subvariants arising from the variant-of-concern (VOC) Omicron include BA.1, BA.1.1, BA.2, BA.2.12.1, BA.4, and BA.5. All possess multiple mutations in their Spike glycoprotein, notably in its immunogenic receptor-binding domain (RBD), and present enhanced viral transmission. The highly mutated Spike glycoproteins from these subvariants present different degrees of resistance to recognition and cross-neutralisation by plasma from previously infected and/or vaccinated individuals. We have recently shown that the temperature affects the interaction between the Spike and its receptor, the angiotensin converting enzyme 2 (ACE2). The affinity of RBD for ACE2 is significantly increased at lower temperatures. However, whether this is also observed with the Spike of Omicron and sub-lineages is not known. Here we show that, similar to other variants, Spikes from Omicron sub-lineages bind better the ACE2 receptor at lower temperatures. Whether this translates into enhanced transmission during the fall and winter seasons remains to be determined.

## 1. Introduction

Severe acute respiratory syndrome coronavirus 2 (SARS-CoV-2) is the causative agent of the coronavirus disease 2019 (COVID-19) pandemic, which still looms over populations worldwide. Since the start of the pandemic, strategies such as vaccination or therapeutic interventions using monoclonal antibodies or antivirals have been used to prevent and control the infection [1]. Amongst them, vaccination remains the only preventative measure and has been proven effective against SARS-CoV-2 infection from earlier variants [2,3,4] and remains effective at protecting from severe outcomes caused by newly emerged variants of concern (VOCs) [5,6,7]. Currently approved vaccines target predominantly the Spike glycoprotein (S), which is responsible for viral entry. The Spike is a trimer comprised of three surface S1 and three transmembrane S2 subunits. The S1 subunit uses its highly immunogenic receptor-binding domain (RBD) to interact with the human angiotensin-converting enzyme 2 (ACE2), following which the S2 subunit mediates viral fusion with the host membrane [8,9,10,11,12]. The structure of the S glycoprotein has been solved by cryo-electron microscopy and X-ray crystallography [9,12,13,14,15], and its dynamic conformational landscape studied by single-molecule Förster resonance energy transfer (smFRET) [16,17,18,19,20,21,22].

Variants of concern (VOCs) denominated by the World Health Organization (WHO) harbour mutations in different regions of the virus, notably in the S glycoprotein since it is under high selective pressure to evade host immune response [23]. The most recent VOC, Omicron (B.1.1.529) and its sub-lineages (BA.1, BA.1.1, BA.2, BA.2.12.1, BA.2.75, BA.4, BA.5), possess over 30 mutations compared to S from ancestral strains and share over 20 mutations between them [24,25,26,27,28,29]. Omicron and its subvariants show enhanced transmissibility and immune evasion from antibodies elicited in vaccinated and previously infected individuals, causing global concern over vaccine failure and immune escape [28,29,30,31,32,33,34,35].

One of the many factors influencing transmissibility appears to be linked to the capacity of the different VOC Spikes to interact with the ACE2 receptor [36,37]. Previous work has shown that this interaction is influenced by temperature [38]. Here, by combining an array of biochemical and biological assays, including flow cytometry, virus capture assay, and biolayer interferometry, we report on the impact that temperature has on the capacity of Omicron subvariant Spikes to interact with human ACE2.

## 2. Materials and Methods

### 2.1. Plasmids

The plasmids expressing SARS-CoV-2 Spike D614G and SARS-CoV-2 S RBD (residues 319–541) fused with a hexahistidine tag were previously described [39]. The plasmids encoding the SARS-CoV-2 S RBD from the B.1.1.529-BA.2 lineage was synthesised commercially by Genscript (Piscataway, NJ, USA). The RBD sequence (encoding for residues 319–541) fused to a C-terminal hexa-histidine tag was cloned into the pcDNA3.1(+) expression vector. The plasmids encoding the full-length *spike* from the B.1.617.2 (Delta), B.1.1.529 (Omicron) BA.1, BA.1.1, BA.2, BA.2.12.1, BA.4/5 lineages were generated by overlapping PCR using a codon-optimised wild-type SARS-CoV-2 *spike* gene that was synthesised (Biobasic, Markham, ON, Canada) and cloned in pCAGGS as a template [16,17,40]. All constructs were validated by Sanger sequencing. The plasmid encoding for soluble human ACE2 (residues 1–615) fused with an 8xHisTag was reported elsewhere [12]. The plasmid encoding for the ACE2-Fc chimeric protein, a protein composed of an ACE2 ectodomain (1–615) linked to an Fc segment of human IgG1, was previously reported [41]. The lentiviral vector pNL4.3 R-E− Luc was obtained from the NIH AIDS Reagent Program. The vesicular stomatitis virus G (VSV-G)-encoding plasmid was previously described [42].

### 2.2. Cell Lines

HEK 293T cells (obtained from the American Type Culture Collection [ATCC]) were derived from 293 cells, into which the simian virus 40 T-antigen was inserted. Cf2Th cells (ATCC) are canine thymocytes resistant to SARS-CoV-2 entry and were used as target cells in the virus capture assay. 293T cells and Cf2Th were maintained at 37 °C under 5% CO_2_ in Dulbecco’s modified Eagle’s medium (DMEM) (Wisent, St. Bruno, QC, Canada), supplemented with 5% fetal bovine serum (FBS) (VWR, Radnor, PA, USA) and 100 U/mL penicillin/streptomycin (Wisent).

### 2.3. Protein Expression and Purification

FreeStyle 293F cells (Invitrogen, Waltham, MA, USA) were grown in FreeStyle 293F medium (Invitrogen) to a density of 1 × 10^6^ cells/mL at 37 °C with 8% CO_2_ with regular agitation (150 rpm). Cells were transfected with a plasmid coding for SARS-CoV-2 Omicron BA.2 S RBD (319-537), soluble ACE2 (sACE2, 1-615), or ACE2-Fc (1-615), using ExpiFectamine 293 transfection reagent, as directed by the manufacturer (Invitrogen). One week later, cells were pelleted and discarded. Supernatants were filtered using a 0.22 µm filter (Thermo Fisher Scientific, Waltham, MA, USA). The recombinant sACE2 protein and RBD proteins were purified by nickel affinity columns, as directed by the manufacturer (Invitrogen) and ACE2-Fc was purified using a protein A affinity column (Cytiva, Marlborough, MA, USA), as directed by the manufacturer. The protein preparations were dialysed against phosphate-buffered saline (PBS) and stored at −80 °C in aliquots until further use. To assess purity, recombinant proteins were loaded on SDS-PAGE gels and stained with Coomassie Blue. Purified proteins were >95% pure after size-exclusion chromatography as verified by SDS-PAGE and Coomassie blue staining.

### 2.4. Flow Cytometry Analysis of Cell-Surface Staining

Using the standard calcium phosphate method, 10 µg of *spike* expressor and 2 µg of a green fluorescent protein (GFP) expressor (pIRES2-GFP, Clontech) were transfected into 2 × 10^6^ 293T cells. At 48 h post transfection, 293T cells were stained with anti-Spike monoclonal antibodies CV3-25 (5 µg/mL) or using the ACE2-Fc chimeric protein (20 µg/mL) for 45 min at 37 °C, 22 °C, or 4 °C. Alternatively, to determine the Hill coefficients, cells were preincubated with increasing concentrations of sACE2 (0 to 665 nM) at 37 °C or 4 °C. sACE2 binding was detected using a polyclonal goat anti-ACE2 (RND systems, Minneapolis, MN, USA). AlexaFluor-647-conjugated goat anti-human IgG (H + L) Ab (Invitrogen) and AlexaFluor-647-conjugated donkey anti-goat IgG (H + L) Ab (Invitrogen) were used as secondary antibodies to stain cells for 30 min at room temperature. The percentage of transfected cells (GFP+ cells) was determined by gating the living cell population based on viability dye staining (Aqua Vivid, Invitrogen). Samples were acquired on an LSRII cytometer (BD Biosciences, Mississauga, ON, Canada) and data analysis was performed using FlowJo v10.3 (Tree Star, Ashland, OR, USA). Hill coefficient analyses were done using GraphPad Prism version 8.0.1 (GraphPad, San Diego, CA, USA).

### 2.5. Virus Capture Assay

The SARS-CoV-2 virus capture assay was previously reported [43]. Pseudoviral particles were produced by transfecting 2 × 10^6^ 293T cells with pNL4.3 R-E− Luc (3.5 μg), plasmids encoding for SARS-CoV-2 Spike (1 μg) proteins and VSV-G (1 μg) using the standard calcium phosphate method. Forty-eight hours later, virus-containing supernatants were collected, and cell debris were removed through centrifugation (1500 rpm for 10 min). The CV3-25 antibody or ACE2-Fc protein was immobilised on white MaxiSorp ELISA plates (Thermo Fisher Scientific) at a concentration of 5 μg/mL in 100 μL of PBS overnight at 4 °C. Unbound proteins were removed by washing the plates twice with PBS. Plates were subsequently blocked with 3% bovine serum albumin (BSA) in PBS for 1 h at room temperature, followed by 1 h incubation at 37 °C, 22 °C, or 4 °C. Meanwhile, virus-containing supernatants were pre-tempered at 37 °C, 22 °C, or 4 °C for 1 h. After washing plates twice with PBS, 200 μL of virus-containing supernatant were added to the wells. After 30 min of incubation at 37 °C, 22 °C, or 4 °C, respectively, supernatants were discarded, and the wells were washed with PBS three times. Virus capture was visualised by adding 1 × 10^4^ SARS-CoV-2-resistant Cf2Th cells per well in complete DMEM. Forty-eight hours post-infection, cells were lysed by the addition of 30 μL of passive lysis buffer (Promega, Madison, WI, USA) and one freeze–thaw cycle. An LB942 TriStar luminometer (Berthold Technologies, Bad Wildbad, Germany) was used to measure the luciferase activity of each well after the addition of 100 μL of luciferin buffer (15 mM MgSO_4_, 15 mM KH_2_PO_4_ [pH 7.8], 1 mM ATP, and 1 mM dithiothreitol) and 50 μL of 1 mM D-luciferin potassium salt (Prolume, Randolph, VT, USA).

### 2.6. Biolayer Interferometry

Binding kinetics were performed with an Octet RED96e system (ForteBio, Fremont, CA, USA) at different temperatures (10 °C, 25 °C, and 35 °C), shaking at 1000 RPM. Amine-reactive second-generation (AR2G) biosensors (Sartorius, Göttingen, Germany) were hydrated in water, then activated for 300 s with a solution of 5 mM sulfo-NHS and 10 mM EDC (Sartorius) prior to amine coupling. Either SARS-CoV-2 RBD WT or BA.2 were loaded into the AR2G biosensor at 12.5 µg/mL at 25 °C in 10 mM acetate solution pH 5 for 600 s then quenched into 1 M ethanolamine solution pH 8.5 (Sartorius) for 300 s. Loaded biosensor were placed in a 10× kinetics buffer (Sartorius) for 120 s for baseline equilibration. Association of sACE2 (in the 10× kinetics buffer) to the different RBD proteins was carried out for 180 s at various concentrations in a twofold dilution series from 500 nM to 31.25 nM prior to dissociation for 300 s. The data were baseline subtracted prior to fitting being performed using a 1:1 binding model and the ForteBio data analysis software. Calculation of on rates (k_on_), off rates (k_off_), and affinity constants (K_D_) was computed using a global fit applied to all data.

### 2.7. Statistical Analysis

Statistical analyses were done using GraphPad Prism version 8.0.1 (GraphPad). Every dataset was tested for statistical normality and this information was used to apply the appropriate (parametric or nonparametric) statistical analysis. Difference in ACE2-Fc recognition and viral entry by VOC full Spikes were analyzed using Mann–Whitney U tests. Outliers were ruled out by Rout’s outlier test (Rout Q = 10%). *p* values < 0.05 were considered significant; significance values are indicated as * *p* < 0.05, ** *p* < 0.01, *** *p* < 0.001, and **** *p* < 0.0001.

## 3. Results

### 3.1. Spike Changes in Delta and Omicron Subvariants Compared to the Ancestral D614G Strain

**Figure 1:**
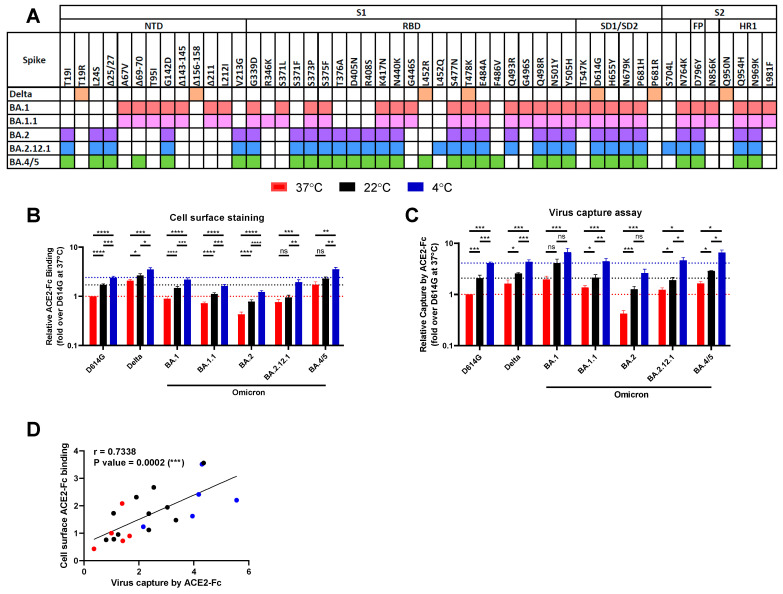
Low temperature enhances Omicron subvariant Spikes’ interactions with ACE2. (**A**) List of point mutations and deletions in the Spike amino acid sequence from Delta variant and Omicron subvariants compared to the D614G ancestral strain. NTD: N-terminal domain; RBD: receptor binding domain; SD1/SD2: subdomains 1 and 2; FP: fusion peptide; HR1: heptad repeat region 1. (**B**,**C**) Cell surface staining of transfected 293T cells and virus capture assay of Omicron and subvariant Spikes at 37 °C (red), 22 °C (black), and 4 °C (blue). (**B**) ACE2-Fc recognition is presented as a ratio of ACE2 binding to D614G Spike obtained at 37 °C. (**C**) Pseudoviruses encoding the luciferase gene (Luc+) and expressing the SARS-CoV-2 Spike D614G, Delta or from Omicron subvariants (BA.1, BA.1.1, BA.2, BA.2.12.1, BA.4/5) were tested for viral capture by ACE2-Fc at the respective temperatures. Relative light units (RLU) obtained using ACE2-Fc was normalised to the signal obtained with the temperature-independent CV3-25 mAb and presented as a ratio of ACE2 capture to D614G obtained at 37 °C. (**D**) Correlation between virus capture and cell surface binding of Spike by ACE2-Fc is depicted for the three temperatures tested. These results represent at least three independent experiments showing means ± SEM. Statistical significance was tested using (**B**,**C**) the Mann–Whitney U test or (**D**) Spearman rank correlation test (* *p* < 0.05; ** *p* < 0.01; *** *p* < 0.001, **** *p* < 0.0001, ns: non-significant).

Omicron (B.1.1.529) subvariants (BA.1, BA.1.1, BA.2, BA.2.12.1, BA.4, and BA.5) Spikes accumulated the larger number of mutations amongst all VOCs to date. They share 20 mutations between them (Figure 1A). We compared the ACE2-binding capacity of different Omicron subvariant Spikes with the ancestral strain D614G and the previous Delta (B.1.617.2) VOC Spike. The original Omicron BA.1 has 13 unique mutations, and its sub-lineage BA.1.1 differs from BA.1 by one mutation (R346K). Compared to BA.1, the BA2 subvariant has eight unique mutations, and harbours entirely different NTD mutations (T19I, L24S, Δ25/27, V213G), sharing only the G142D substitution. In the RBD, BA.2 shares 12 mutations with BA.1 but shows one different substitution at residue 371 (S371F; S371L in BA.1) and three extra mutations, T376A, D405N, and R408S. The BA.2.12.1 sub-lineage harbours the same 29 mutations than BA.2, with two extra mutations, L452Q in the RBD and S704L in the S2 subunit. The BA.4 and BA.5 subvariants are derived from BA.2, differing by one deletion (Δ69-70), one reversion (R493Q), and two additional mutations (L452R and F486V). The RBD of the Omicron strains have over 14 extra mutations compared to the VOC with the highest ACE2 affinity, Alpha (B.1.1.7), which had only one RBD mutation (N501Y) [44]. The T478K mutation is shared between the Delta variant and all Omicron subvariants. BA.4/5 shares the L452R mutation with Delta, a mutation that enhances ACE2 interaction [44].

### 3.2. Temperature Modulation of D614G, Delta and Omicron Spikes Interaction with ACE2

We first measured ACE2 recognition of Omicron subvariants using a flow cytometry assay, as described [38,44]. Briefly, plasmids expressing the full-length SARS-CoV-2 Spike of the ancestral strain D614G, the previously prominent VOC Delta, and current Omicron subvariants were transfected in HEK 293T cells. ACE2 interaction was determined by using the chimeric ACE2-Fc protein, which is composed of two ACE2 ectodomains linked to the Fc portion of human IgG [41].

To ensure that any differential recognition was not linked to a temperature-dependent variation in Spike levels, we used the conformational- and temperature-independent S2-targeting monoclonal antibody (mAb) CV3-25 as an experimental control (Figure S1) [19,38,40,45]. Compared to the ancestral D614G Spike, the Delta and BA.4/5 Spikes presented an increase in ACE2-Fc interaction at 37 °C whereas BA.1.1, BA.2, and BA.2.12.1 bound ACE2 less efficiently (Figure 1B). At this temperature, we observed no differences with BA.1.

Previous studies have reported that lower temperatures enhance RBD affinity for ACE2 and favour the adoption of the “up” conformation, therefore enhancing the capacity of the trimeric Spike to interact with ACE2 [38,44,45]. We therefore evaluated whether this was the case for the Omicron Spike and its subvariants. Forty-eight hours post-transfection, Spike-expressing cells were incubated at different temperatures (37 °C, 22 °C, and 4 °C) before measuring ACE2-Fc binding by flow cytometry, as described above. We observed a gradual increase in ACE2-Fc binding concomitant with the temperature decrease for all Spikes tested (Figure 1B), validating a temperature-dependent interaction between Spike and ACE2 [38,44].

We then evaluated whether the observed increase in Spike–ACE2 interaction at low temperature was maintained when the Spike was expressed at the surface of pseudoviral particles. To this end, we used a previously described virus capture assay [43] that uses pseudoviral particles bearing the different SARS-CoV-2 Spikes and evaluated their ability to interact with ACE2-Fc immobilised on ELISA plates. In agreement with a better interaction with ACE2 at lower temperatures, we observed a stepwise increase in viral capture at colder temperatures for all Spikes tested (Figure 1C), which significantly correlated with the cell-based binding assay (Figure 1D). Thus, our findings indicate that the Spike–ACE2 interaction is similarly modulated by temperature independently of whether the Spike is expressed on viral particles or cell membranes.

### 3.3. Temperature Modulates ACE2 Binding Cooperativity and Affinity for Omicron Spikes

**Figure 2:**
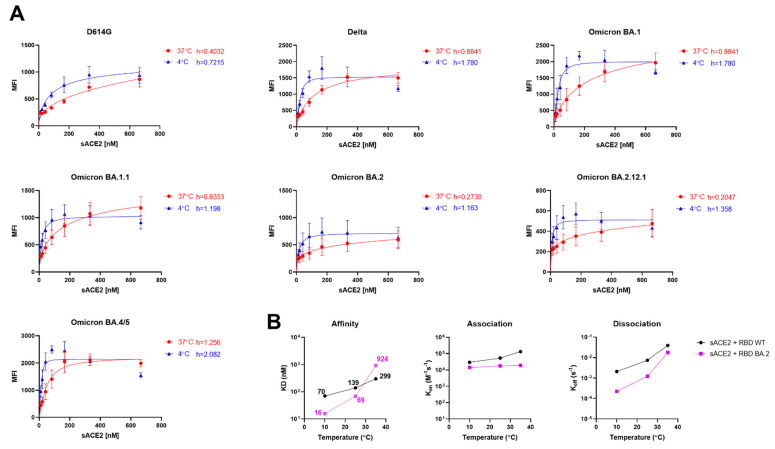
Low temperatures “open” Omicron subvariant Spikes. (**A**) The binding of sACE2 to the Spike of D614G, Delta, Omicron BA.1, BA.1.1, BA.2, BA.2.12.1, BA.4/5 expressed on the surface of 293T cells was measured by flow cytometry. Increasing concentrations of sACE2 were incubated with Spike-expressing cells in 4 °C (blue) or 37 °C (red). Means ± SEM derived from at least three independent experiments are shown. The Hill coefficients were calculated using GraphPad software. (**B**) Binding kinetics between SARS-CoV-2 RBD (WT or BA.2) and sACE2 assessed by biolayer interferometry at different temperatures. Graphs represent the affinity constants (K_D_), on rates (K_on_), and off rates (K_off_) values obtained at different temperatures and calculated using a 1:1 binding model. Raw data and fitting models are presented in Figure S2.

The Spike interacts with its ACE2 receptor in its “up” conformation [46]. However, the Spike trimer of Omicron BA.1 and subvariant BA.2 was reported to assume more the RBD “down” conformation that is stabilised by a strong network of inter-protomer contacts leading to its higher thermostability [47,48]. Therefore, we studied the sensitivity of the Spike subvariants to conformational changes in response to ACE2. To do so, we calculated the Hill coefficient (h), which is the degree of binding cooperativity between the protomers of the trimeric Spike and monomeric ACE2 molecules in a concentration-response manner. The h values are calculated from the steepness of dose-response curves generated upon incubation of Spike-expressing cells with increasing concentrations of sACE2, as previously described [38]. Briefly, HEK293T cells were transfected with full-length Spikes from the D614G ancestral strain, the Delta VOC or the Omicron subvariants. With the exception of BA.4/ 5, all other Spikes tested presented a negative cooperativity (h value < 1) at 37 °C (Figure 2A, red lines). This is consistent with previous observations suggesting an energetic barrier to engage additional ACE2 molecules at high temperatures [38]. The Spike from BA.4/5 presented a positive Hill coefficient (h = 1.256) at 37 °C, thus suggesting a coordinated Spike opening at warmer temperatures (Figure 2A). Interestingly, sACE2 binding cooperativity was improved in all Spikes at low temperature (4 °C), confirming that low temperatures facilitate ACE2-induced Spike opening (Figure 2A, blue lines). Interestingly, the ACE2 binding cooperativity with BA.4/5 Spike at 37 °C (h = 1.256) was found to be similar to its parental BA.2 lineage at 4 °C (h = 1.163), suggesting a lesser reliability on cold temperatures to expose RBD in the “up” conformation.

To assess the temperature’s role in modulating binding kinetics between Omicron RBD and ACE2, we performed biolayer interferometry (BLI) experiments at different temperatures (10 °C, 25 °C, and 35 °C) (Figure 2B). We observed a drastic decrease in the off rate at lower temperatures compared with its wild-type (WT, Wuhan-Hu-1 strain) counterpart. As observed previously [38,44], the affinity of the RBD with its receptor is mainly dictated by its off rate as the RBD BA.2 has a 4.5-fold decrease in K_D_ compared to WT at colder temperatures.

## 4. Discussion

Previous studies have shown that low temperature impacts the conformation of the Spike, triggering trimer opening and increasing binding to the ACE2 receptor [38,44,45]. Moreover, we have previously shown that this translates into enhanced viral attachment and infection [38]. Indeed, we observed enhanced infection at low temperatures using pseudoviral particles, as well as authentic SARS-CoV-2, the latter with primary human airway epithelial cells as target cells [38]. Since Omicron harbours 33 mutations in its Spike glycoprotein with mutations in the S2 stabilizing the RBD “down” conformation [47], we wondered whether these mutations hindered the impact of low temperature on Spike conformation and ACE2 interaction.

In this study, we investigated if temperature affects the interactions between SARS-CoV-2 Omicron subvariant Spikes and the primary receptor ACE2 in vitro with ELISA and flow cytometry assays. We have shown that the affinity and binding of Omicron subvariant Spikes to ACE2 receptors are significantly enhanced at low temperatures in cells and pseudoviral particles expressing the different Spikes. Importantly, our results show that low temperature facilitates the capacity of all Omicron subvariant Spikes to interact with their receptors, which could be explained by enhanced cooperativity between protomers upon ACE2 interaction and slower off-rate.

Our results suggest that even VOC Spikes that have a more “closed” conformation can be affected by low temperatures, enhancing binding to the ACE2 receptor. Previous VOCs such as Alpha and Delta only possess a few mutations that impact antibody recognition and ACE2 binding. However, with the widespread vaccination and infection of individuals, a stronger immune pressure over the virus emerged [23], leading to the appearance of highly mutated Spike variants [49]. Omicron and its subvariants have evolved to escape immune pressure [50,51]. However, some mutations appear to have been also selected for improved ACE2 interaction with BA.4/5 harbouring the L452R mutation, well-known to increase affinity for ACE2 while helping to evade immune responses [52].

Numerous studies offered strong evidence that temperature was a significant factor that can impact the aerosol transmission of SARS-CoV-2 [53]. How the improved Spike–ACE2 interaction at lower temperatures described in this manuscript affects viral transmission remains unknown. Further experiments in animal models will be required to address this question. However, with the constant evolution and selection of mutations leading to an increase in transmissibility and antigenic shift as seen for the Omicron lineage [54,55], it remains crucial to continue to study how these new selected mutations impact the interaction between the SARS-CoV-2 Spike and its human receptor, but also how temperature affects this interaction.

In summary, our results show that Omicron and its subvariant are sensitive to the effect of low temperature, though it is unclear whether this mechanism contributes to viral transmission or to the seasonality of VOCs. Our study indicates that the Spike–ACE2 affinity needs to be considered when evaluating the effect of temperature on SARS-CoV-2 transmission.

## Data Availability

Data are contained within the article and supplementary materials.

## Supplementary Materials

The following supporting information can be downloaded at https://www.mdpi.com/article/10.3390/v14102178/s1. Figure S1. Temperature does not affect SARS-CoV-2 Omicron Spikes cell surface expression, Figure S2. Enhanced affinity of SARS-CoV-2 RBD BA.2 for ACE2 at low temperatures.

## References

[1] Zhang C., Yang M. (2022). Newly Emerged Antiviral Strategies for SARS-CoV-2: From Deciphering Viral Protein Structural Function to the Development of Vaccines, Antibodies, and Small Molecules. Int. J. Mol. Sci..

[2] Polack F.P., Thomas S.J., Kitchin N., Absalon J., Gurtman A., Lockhart S., Perez J.L., Pérez Marc G., Moreira E.D., Zerbini C. (2020). Safety and Efficacy of the BNT162b2 mRNA Covid-19 Vaccine. N. Engl. J. Med..

[3] Baden L.R., El Sahly H.M., Essink B., Kotloff K., Frey S., Novak R., Diemert D., Spector S.A., Rouphael N., Creech C.B. (2020). Efficacy and Safety of the mRNA-1273 SARS-CoV-2 Vaccine. N. Engl. J. Med..

[4] Sadoff J., Gray G., Vandebosch A., Cárdenas V., Shukarev G., Grinsztejn B., Goepfert P.A., Truyers C., Fennema H., Spiessens B. (2021). Safety and Efficacy of Single-Dose Ad26.COV2.S Vaccine against Covid-19. N. Engl. J. Med..

[5] Ionescu I.G., Skowronski D.M., Sauvageau C., Chuang E., Ouakki M., Kim S., De Serres G. (2022). BNT162b2 effectiveness against Delta & Omicron variants in teens by dosing interval and duration. medRxiv.

[6] Kislaya I., Casaca P., Borges V., Sousa C., Ferreira B.I., Fernandes E., Dias C.M., Duarte S., Almeida J.P., Grenho I. (2022). SARS-CoV-2 BA.5 vaccine breakthrough risk and severity compared with BA.2: A case-case and cohort study using Electronic Health Records in Portugal. medRxiv.

[7] Camacho J., Giménez E., Albert E., Zulaica J., Álvarez-Rodríguez B., Torres I., Rusu L., Burgos J.S., Peiró S., Vanaclocha H. (2022). Cumulative incidence of SARS-CoV-2 infection in the general population of the Valencian Community (Spain) after the surge of the Omicron BA.1 variant. medRxiv.

[8] Hoffmann M., Kleine-Weber H., Schroeder S., Kruger N., Herrler T., Erichsen S., Schiergens T.S., Herrler G., Wu N.H., Nitsche A. (2020). SARS-CoV-2 Cell Entry Depends on ACE2 and TMPRSS2 and Is Blocked by a Clinically Proven Protease Inhibitor. Cell.

[9] Shang J., Ye G., Shi K., Wan Y., Luo C., Aihara H., Geng Q., Auerbach A., Li F. (2020). Structural basis of receptor recognition by SARS-CoV-2. Nature.

[10] Walls A.C., Xiong X., Park Y.J., Tortorici M.A., Snijder J., Quispe J., Cameroni E., Gopal R., Dai M., Lanzavecchia A. (2019). Unexpected Receptor Functional Mimicry Elucidates Activation of Coronavirus Fusion. Cell.

[11] Walls A.C., Park Y.J., Tortorici M.A., Wall A., McGuire A.T., Veesler D. (2020). Structure, Function, and Antigenicity of the SARS-CoV-2 Spike Glycoprotein. Cell.

[12] Wrapp D., Wang N., Corbett K.S., Goldsmith J.A., Hsieh C.L., Abiona O., Graham B.S., McLellan J.S. (2020). Cryo-EM structure of the 2019-nCoV spike in the prefusion conformation. Science.

[13] Cai Y., Zhang J., Xiao T., Peng H., Sterling S.M., Walsh R.M., Rawson S., Rits-Volloch S., Chen B. (2020). Distinct conformational states of SARS-CoV-2 spike protein. Science.

[14] Lan J., Ge J., Yu J., Shan S., Zhou H., Fan S., Zhang Q., Shi X., Wang Q., Zhang L. (2020). Structure of the SARS-CoV-2 spike receptor-binding domain bound to the ACE2 receptor. Nature.

[15] Yan R., Zhang Y., Li Y., Xia L., Guo Y., Zhou Q. (2020). Structural basis for the recognition of SARS-CoV-2 by full-length human ACE2. Science.

[16] Li W., Chen Y., Prévost J., Ullah I., Lu M., Gong S.Y., Tauzin A., Gasser R., Vézina D., Anand S.P. (2021). Structural basis and mode of action for two broadly neutralizing antibodies against SARS-CoV-2 emerging variants of concern. Cell Rep..

[17] Ding S., Ullah I., Gong S.Y., Grover J.R., Mohammadi M., Chen Y., Vézina D., Beaudoin-Bussières G., Verma V.T., Goyette G. (2022). VE607 stabilizes SARS-CoV-2 Spike in the “RBD-up” conformation and inhibits viral entry. iScience.

[18] Lu M., Uchil P.D., Li W., Zheng D., Terry D.S., Gorman J., Shi W., Zhang B., Zhou T., Ding S. (2020). Real-Time Conformational Dynamics of SARS-CoV-2 Spikes on Virus Particles. Cell Host Microbe.

[19] Ullah I., Prevost J., Ladinsky M.S., Stone H., Lu M., Anand S.P., Beaudoin-Bussieres G., Symmes K., Benlarbi M., Ding S. (2021). Live imaging of SARS-CoV-2 infection in mice reveals that neutralizing antibodies require Fc function for optimal efficacy. Immunity.

[20] Yang Z., Han Y., Ding S., Shi W., Zhou T., Finzi A., Kwong P.D., Mothes W., Lu M. (2022). SARS-CoV-2 Variants Increase Kinetic Stability of Open Spike Conformations as an Evolutionary Strategy. mBio.

[21] Guo H., Gao Y., Li T., Li T., Lu Y., Zheng L., Liu Y., Yang T., Luo F., Song S. (2022). Structures of Omicron spike complexes and implications for neutralizing antibody development. Cell Rep..

[22] Diaz-Salinas M.A., Li Q., Ejemel M., Yurkovetskiy L., Luban J., Shen K., Wang Y., Munro J.B. (2022). Conformational dynamics and allosteric modulation of the SARS-CoV-2 spike. eLife.

[23] Prévost J., Finzi A. (2021). The great escape? SARS-CoV-2 variants evading neutralizing responses. Cell Host Microbe.

[24] CDC (2022). COVID Data Tracker—Variant Proportions. https://covid.cdc.gov/covid-data-tracker/#datatracker-home.

[25] Viana R., Moyo S., Amoako D.G., Tegally H., Scheepers C., Althaus C.L., Anyaneji U.J., Bester P.A., Boni M.F., Chand M. (2022). Rapid epidemic expansion of the SARS-CoV-2 Omicron variant in southern Africa. Nature.

[26] Gangavarapu K., Latif A.A., Mullen J.L., Alkuzweny M., Hufbauer E., Tsueng G., Haag E., Zeller M., Aceves C.M., Zaiets K. (2022). Outbreak.info genomic reports: Scalable and dynamic surveillance of SARS-CoV-2 variants and mutations. medRxiv.

[27] Tsueng G., Mullen J.L., Alkuzweny M., Cano M., Rush B., Haag E., Outbreak C., Latif A.A., Zhou X., Qian Z. (2022). Outbreak.info Research Library: A standardized, searchable platform to discover and explore COVID-19 resources. bioRxiv.

[28] Bowen J.E., Addetia A., Dang H.V., Stewart C., Brown J.T., Sharkey W.K., Sprouse K.R., Walls A.C., Mazzitelli I.G., Logue J.K. (2022). Omicron spike function and neutralizing activity elicited by a comprehensive panel of vaccines. Science.

[29] Hachmann N.P., Miller J., Collier A.Y., Ventura J.D., Yu J., Rowe M., Bondzie E.A., Powers O., Surve N., Hall K. (2022). Neutralization Escape by SARS-CoV-2 Omicron Subvariants BA.2.12.1, BA.4, and BA.5. N. Engl. J. Med..

[30] Dejnirattisai W., Huo J., Zhou D., Zahradník J., Supasa P., Liu C., Duyvesteyn H.M.E., Ginn H.M., Mentzer A.J., Tuekprakhon A. (2022). SARS-CoV-2 Omicron-B.1.1.529 leads to widespread escape from neutralizing antibody responses. Cell.

[31] Gobeil S.M., Henderson R., Stalls V., Janowska K., Huang X., May A., Speakman M., Beaudoin E., Manne K., Li D. (2022). Structural diversity of the SARS-CoV-2 Omicron spike. Mol. Cell.

[32] McCallum M., Czudnochowski N., Rosen Laura E., Zepeda Samantha K., Bowen John E., Walls Alexandra C., Hauser K., Joshi A., Stewart C., Dillen Josh R. (2022). Structural basis of SARS-CoV-2 Omicron immune evasion and receptor engagement. Science.

[33] Wang Q., Anang S., Iketani S., Guo Y., Liu L., Katsamba P.S., Shapiro L., Ho D.D., Sodroski J.G. (2022). Functional properties of the spike glycoprotein of the emerging SARS-CoV-2 variant B.1.1.529. Cell Rep..

[34] Qu P., Faraone J., Evans J.P., Zou X., Zheng Y.M., Carlin C., Bednash J.S., Lozanski G., Mallampalli R.K., Saif L.J. (2022). Neutralization of the SARS-CoV-2 Omicron BA.4/5 and BA.2.12.1 Subvariants. N. Engl. J. Med..

[35] Evans J.P., Zeng C., Qu P., Faraone J., Zheng Y.M., Carlin C., Bednash J.S., Zhou T., Lozanski G., Mallampalli R. (2022). Neutralization of SARS-CoV-2 Omicron sub-lineages BA.1, BA.1.1, and BA.2. Cell Host Microbe.

[36] Kuba K., Yamaguchi T., Penninger J.M. (2021). Angiotensin-Converting Enzyme 2 (ACE2) in the Pathogenesis of ARDS in COVID-19. Front. Immunol..

[37] Tortorici M.A., Veesler D. (2019). Structural insights into coronavirus entry. Adv. Virus Res..

[38] Prévost J., Richard J., Gasser R., Ding S., Fage C., Anand S.P., Adam D., Gupta Vergara N., Tauzin A., Benlarbi M. (2021). Impact of temperature on the affinity of SARS-CoV-2 Spike glycoprotein for host ACE2. J. Biol. Chem..

[39] Beaudoin-Bussieres G., Laumaea A., Anand S.P., Prevost J., Gasser R., Goyette G., Medjahed H., Perreault J., Tremblay T., Lewin A. (2020). Decline of Humoral Responses against SARS-CoV-2 Spike in Convalescent Individuals. mBio.

[40] Chatterjee D., Tauzin A., Marchitto L., Gong S.Y., Boutin M., Bourassa C., Beaudoin-Bussières G., Bo Y., Ding S., Laumaea A. (2022). SARS-CoV-2 Omicron Spike recognition by plasma from individuals receiving BNT162b2 mRNA vaccination with a 16-week interval between doses. Cell Rep..

[41] Anand S.P., Chen Y., Prevost J., Gasser R., Beaudoin-Bussieres G., Abrams C.F., Pazgier M., Finzi A. (2020). Interaction of Human ACE2 to Membrane-Bound SARS-CoV-1 and SARS-CoV-2 S Glycoproteins. Viruses.

[42] Emi N., Friedmann T., Yee J.K. (1991). Pseudotype formation of murine leukemia virus with the G protein of vesicular stomatitis virus. J. Virol..

[43] Ding S., Laumaea A., Benlarbi M., Beaudoin-Bussières G., Gasser R., Medjahed H., Pancera M., Stamatatos L., McGuire A.T., Bazin R. (2020). Antibody Binding to SARS-CoV-2 S Glycoprotein Correlates with but Does Not Predict Neutralization. Viruses.

[44] Gong S.Y., Chatterjee D., Richard J., Prévost J., Tauzin A., Gasser R., Bo Y., Vézina D., Goyette G., Gendron-Lepage G. (2021). Contribution of single mutations to selected SARS-CoV-2 emerging variants spike antigenicity. Virology.

[45] Costello S.M., Shoemaker S.R., Hobbs H.T., Nguyen A.W., Hsieh C.L., Maynard J.A., McLellan J.S., Pak J.E., Marqusee S. (2022). The SARS-CoV-2 spike reversibly samples an open-trimer conformation exposing novel epitopes. Nat. Struct. Mol. Biol..

[46] Henderson R., Edwards R.J., Mansouri K., Janowska K., Stalls V., Gobeil S.M.C., Kopp M., Li D., Parks R., Hsu A.L. (2020). Controlling the SARS-CoV-2 spike glycoprotein conformation. Nat. Struct. Mol. Biol..

[47] Stalls V., Lindenberger J., Gobeil S.M., Henderson R., Parks R., Barr M., Deyton M., Martin M., Janowska K., Huang X. (2022). Cryo-EM structures of SARS-CoV-2 Omicron BA.2 spike. Cell Rep..

[48] Zhao Z., Zhou J., Tian M., Huang M., Liu S., Xie Y., Han P., Bai C., Han P., Zheng A. (2022). Omicron SARS-CoV-2 mutations stabilize spike up-RBD conformation and lead to a non-RBM-binding monoclonal antibody escape. Nat. Commun..

[49] Yamasoba D., Kimura I., Nasser H., Morioka Y., Nao N., Ito J., Uriu K., Tsuda M., Zahradnik J., Shirakawa K. (2022). Virological characteristics of the SARS-CoV-2 Omicron BA.2 spike. Cell.

[50] Planas D., Saunders N., Maes P., Guivel-Benhassine F., Planchais C., Buchrieser J., Bolland W.H., Porrot F., Staropoli I., Lemoine F. (2022). Considerable escape of SARS-CoV-2 Omicron to antibody neutralization. Nature.

[51] Liu C., Ginn H.M., Dejnirattisai W., Supasa P., Wang B., Tuekprakhon A., Nutalai R., Zhou D., Mentzer A.J., Zhao Y. (2021). Reduced neutralization of SARS-CoV-2 B.1.617 by vaccine and convalescent serum. Cell.

[52] Deng X., Garcia-Knight M.A., Khalid M.M., Servellita V., Wang C., Morris M.K., Sotomayor-González A., Glasner D.R., Reyes K.R., Gliwa A.S. (2021). Transmission, infectivity, and neutralization of a spike L452R SARS-CoV-2 variant. Cell.

[53] Asif Z., Chen Z., Stranges S., Zhao X., Sadiq R., Olea-Popelka F., Peng C., Haghighat F., Yu T. (2022). Dynamics of SARS-CoV-2 spreading under the influence of environmental factors and strategies to tackle the pandemic: A systematic review. Sustain. Cities Soc..

[54] Shrestha L.B., Foster C., Rawlinson W., Tedla N., Bull R.A. (2022). Evolution of the SARS-CoV-2 omicron variants BA.1 to BA.5: Implications for immune escape and transmission. Rev. Med. Virol..

[55] Telenti A., Hodcroft E.B., Robertson D.L. (2022). The Evolution and Biology of SARS-CoV-2 Variants. Cold Spring Harb. Perspect. Med..

