# Supplementary material for: Temperature Influences the Interaction between SARS-CoV-2 Spike from Omicron Subvariants and Human ACE2
Source: Viruses. 2022 Sep 30;14(10):2178. doi: 10.3390/v14102178 (PMC9607596; doi:10.3390/v14102178)
Supplement: Supplementary file 1 [file viruses-14-02178-s001.zip › viruses-1920545-supplementary.pdf]

Supplementary materials

Temperature influences the interaction between SARS-CoV-2 Spike from Omicron subvariants and human ACE2

Shang Yu Gong, Shilei Ding, Mehdi Benlarbi, Yaozong Chen, Dani Vézina, Lorie Marchitto, Guillaume Beaudoin-Bussières, Guillaume Goyette, Catherine Bourassa, Yuxia Bo, Halima Medjahed, Inès Levade, Marzena Pazgier, Marceline Côté, Jonathan Richard, Jérémie Prévost and Andrés Finzi

<u>**List of included material**</u>

Figure S1. Temperature does not affect SARS-CoV-2 Omicron Spikes cell surface expression.

Figure S2. Enhanced affinity of SARS-CoV-2 RBD BA.2 for ACE2 at low temperatures.

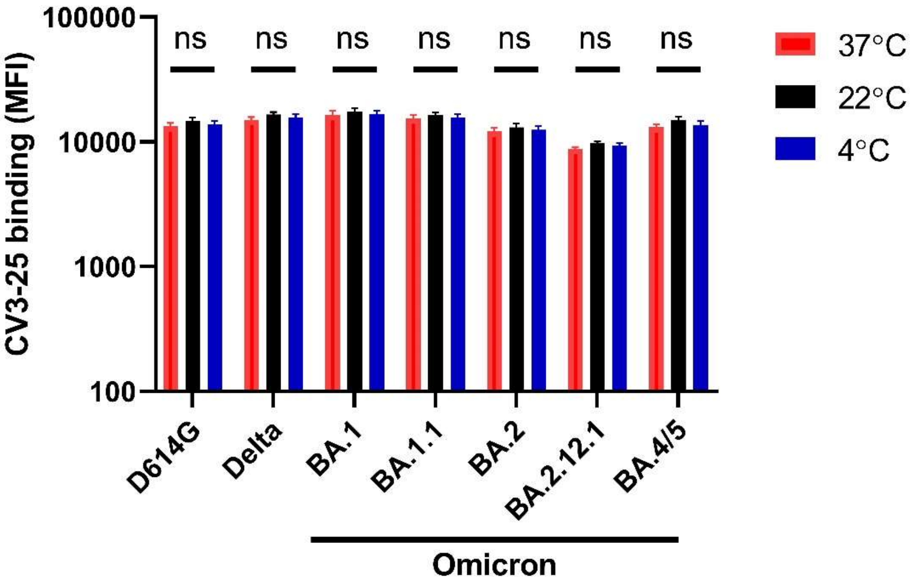

Figure S1. Temperature does not affect SARS-CoV-2 Omicron Spikes cell surface expression. Cell surface staining of 293T cells expressing full length SARS-CoV-2 Spike glycoproteins from indicated variants (D614G, Delta and Omicron subvariants). Conformation and temperature-independent CV3-25 mAb is used to quantify the amount of Spike expressed on the cell surface. The graph presents the median flurescence intensities (MFI). Error bars indicate means ± SEM. These results were obtained in at least three independent experiments. Statistical significance was tested using Mann-Whitney U test (ns, non-significant).

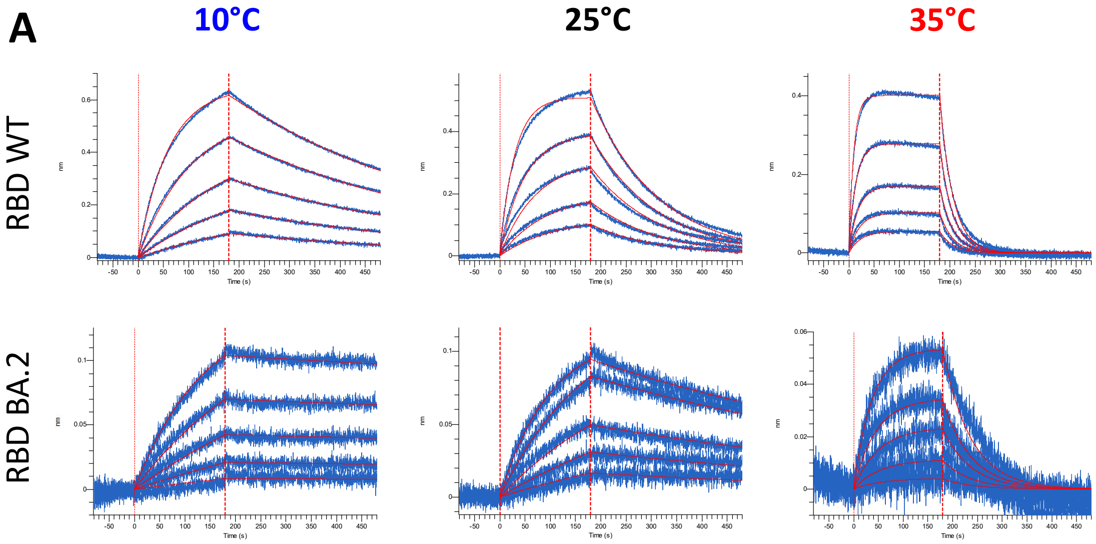

B

sample	Temperature (ºC)	K_D (nM)	K_{on} (1/Ms)	K_{off} (1/s)
sACE2 + RBD WT	10	70	2.98×10^4	2.09×10^{-3}
	25	139	5.41×10^4	7.51×10^{-3}
	35	299	1.31×10^5	3.93×10^{-2}
sACE2 + RBD BA.2	10	16	1.43×10^4	2.23×10^{-4}
	25	69	1.80×10^4	1.24×10^{-3}
	35	924	1.92×10^4	1.78×10^{-2}

Figure S2. Enhanced affinity of SARS-CoV-2 RBD BA.2 for ACE2 at low temperatures. Binding kinetics between SARS-CoV-2 RBD (WT or BA.2) and soluble ACE2 (sACE2) was assessed by BLI at different temperatures. BLI was carried out for 180s at various concentrations in a two-fold dilution series from 500nM to 31.25 nM prior to dissociation for 300 s for BA.2 or WT RBD at 35°C, 25°C, or 10°C respectively. Curve fitting was performed using a 1:1 binding model in the ForteBio data analysis software. Calculation of on-rates (K_{on}), off-rates (K_{off}), and affinity constants (K_D) was computed using a global fit applied to all data. Raw data are presented in blue and fitting models are in red.
